# Abortive Treatment of Whitlow

**Published:** 1889-09

**Authors:** 


### Abortive Treatment of Whitlow.

The Paris correspondent of the *Journal of the American Medical Association*, May 25, 1889, says that Dr. Gaucher, in writing on the abortive treatment of whitlow, states that, to effect this object, it is sufficient to moisten slightly the painful part and around it with a little water, and to pass over this surface a stick of nitrate of silver. In a few hours after the skin becomes black, all pain disappears, and the inflammation is arrested. No dressing is required, and the black color disappears in six days. The author relates that in a case of a fit of the gout, the great toe was much swollen and painful to the touch, rather red, and was the seat of lancinating pains which prevented the patient from sleeping. The toe was painted as above described, and the next day it diminished in size, the pain completely disappeared a quarter of an hour after the painting, and the patient got up and attended to his occupation.

